# PROTOCOL: Barriers and facilitators to stakeholder engagement in health guideline development: A qualitative evidence synthesis

**DOI:** 10.1002/cl2.1237

**Published:** 2022-04-25

**Authors:** Olivia Magwood, Alison Riddle, Jennifer Petkovic, Lyubov Lytvyn, Joanne Khabsa, Pearl Atwere, Elie A. Akl, Pauline Campbell, Vivian Welch, Maureen Smith, Reem A. Mustafa, Heather Limburg, Leonila F. Dans, Nicole Skoetz, Sean Grant, Tom Concannon, Peter Tugwell

**Affiliations:** ^1^ C.T. Lamont Primary Health Care Research Centre Bruyere Research Institute Ottawa Canada; ^2^ School of Epidemiology and Public Health, Faculty of Medicine University of Ottawa Ottawa Canada; ^3^ Bruyère Research Institute University of Ottawa Ottawa Canada; ^4^ Department of Clinical Epidemiology and Biostatistics McMaster University Hamilton Canada; ^5^ Clinical Research Institute American University of Beirut Medical Center Beirut Lebanon; ^6^ Department of Internal Medicine American University of Beirut Medical Center Beirut Lebanon; ^7^ Nursing, Midwifery and Allied Health Professions Research Unit Glasgow Caledonian University Glasgow UK; ^8^ Methods Centre Bruyère Research Institute Ottawa Canada; ^9^ Cochrane Consumer Network Executive Cochrane Ottawa Canada; ^10^ Global Health and Guidelines Division Public Health Agency of Canada Ottawa Canada; ^11^ Department of Clinical Epidemiology University of the Philippines College of Medicine Manilla Philippines; ^12^ Cochrane Cancer, Department I of Internal Medicine, Center for Integrated Oncology Aachen Bonn Cologne Duesseldorf, Faculty of Medicine and University Hospital Cologne University of Cologne Cologne Germany; ^13^ Department of Social and Behavioral Sciences, Richard M. Fairbanks School of Public Health Indiana University Indianapolis Indiana USA; ^14^ The RAND Corporation Boston Massachusetts USA; ^15^ Clinical Epidemiology Program Ottawa Hospital Research Institute Ottawa Canada

## Abstract

**Background:**

There is a need for the development of comprehensive, global, evidence‐based guidance for stakeholder engagement in guideline development. Stakeholders are any individual or group who is responsible for or affected by health‐ and healthcare‐related decisions. This includes patients, the public, providers of health care and policymakers for example. As part of the guidance development process, Multi‐Stakeholder Engagement (MuSE) Consortium set out to conduct four concurrent systematic reviews to summarise the evidence on: (1) existing guidance for stakeholder engagement in guideline development, (2) barriers and facilitators to stakeholder engagement in guideline development, (3) managing conflicts of interest in stakeholder engagement in guideline development and (4) measuring the impact of stakeholder engagement in guideline development. This protocol addresses the second systematic review in the series.

**Objectives:**

The objective of this review is to identify and synthesise the existing evidence on barriers and facilitators to stakeholder engagement in health guideline development. We will address this objective through two research questions: (1) What are the barriers to multi‐stakeholder engagement in health guideline development across any of the 18 steps of the GIN‐McMaster checklist? (2) What are the facilitators to multi‐stakeholder engagement in health guideline development across any of the 18 steps of the GIN‐McMaster checklist?

**Search Methods:**

A comprehensive search strategy will be developed and peer‐reviewed in consultation with a medical librarian. We will search the following databases: MEDLINE, Cumulative Index to Nursing & Allied Health Literature (CINAHL), EMBASE, PsycInfo, Scopus, and Sociological Abstracts. To identify grey literature, we will search the websites of agencies who actively engage stakeholder groups such as the AHRQ, Canadian Institutes of Health Research (CIHR) Strategy for Patient‐Oriented Research (SPOR), INVOLVE, the National Institute for Health and Care Excellence (NICE) and the PCORI. We will also search the websites of guideline‐producing agencies, such as the American Academy of Pediatrics, Australia's National Health Medical Research Council (NHMRC) and the WHO. We will invite members of the team to suggest grey literature sources and we plan to broaden the search by soliciting suggestions via social media, such as Twitter.

**Selection Criteria:**

We will include empirical qualitative and mixed‐method primary research studies which qualitatively report on the barriers or facilitators to stakeholder engagement in health guideline development. The population of interest is stakeholders in health guideline development. Building on previous work, we have identified 13 types of stakeholders whose input can enhance the relevance and uptake of guidelines: Patients, caregivers and patient advocates; Public; Providers of health care; Payers of health services; Payers of research; Policy makers; Program managers; Product makers; Purchasers; Principal investigators and their research teams; and Peer‐review editors/publishers. Eligible studies must describe stakeholder engagement at any of the following steps of the GIN‐McMaster Checklist for Guideline Development.

**Data Collection and Analysis:**

All identified citations from electronic databases will be imported into Covidence software for screening and selection. Documents identified through our grey literature search will be managed and screened using an Excel spreadsheet. A two‐part study selection process will be used for all identified citations: (1) a title and abstract review and (2) full‐text review. At each stage, teams of two review authors will independently assess all potential studies in duplicate using a priori inclusion and exclusion criteria. Data will be extracted by two review authors independently and in duplicate according to a standardised data extraction form.

**Main Results:**

The results of this review will be used to inform the development of guidance for multi‐stakeholder engagement in guideline development and implementation. This guidance will be official GRADE (Grading of Recommendations Assessment, Development and Evaluation) Working Group guidance. The GRADE system is internationally recognised as a standard for guideline development. The findings of this review will assist organisations who develop healthcare, public health and health policy guidelines, such as the World Health Organization, to involve multiple stakeholders in the guideline development process to ensure the development of relevant, high quality and transparent guidelines.

## 1 BACKGROUND

### 1.1 The problem, condition or issue

Health guidelines are ‘systematically developed evidence‐based statements which assist providers, recipients and other stakeholders in making informed decisions about appropriate health interventions’ (WHO, 2003). Guidelines play a crucial role in the delivery of evidence‐based medicine. The systematic examination of evidence promoted by evidence‐based medicine is done through a comprehensive search of the literature, critical appraisal of the quality of the evidence and interpretation of the findings in light of patients' preferences and societal values (Guyatt, 2008). These guidelines assist health practitioners, patients, caregivers, policymakers and other stakeholders to make informed decisions about health practice, public health and health policy. It is increasingly recognised that the engagement of multiple stakeholders in guideline development improves recommendation relevancy, uptake, implementation and sustainability (Esmail, 2015; Gagliardi, 2012; Moulding, 1999).

Stakeholders are ‘any individual or group who is responsible for or affected by health‐ and healthcare‐related decisions that can be informed by research evidence’ (Concannon, 2012). This broad definition includes, but is not limited to, patients, caregivers, the public, patient advocates, providers (e.g., nurses), policymakers (e.g., health ministries), payers of health services (e.g., insurers, public payers) and health research (e.g., national research institutes), purchasers (e.g., employers), researchers, product makers (e.g., drug makers) and peer review editors. Several entities, including the World Health Organization (WHO), the Canadian Institute for Health Research (CIHR), the National Institute for Health and Care Excellence (NICE) and the Grading of Recommendations, Assessment, Development and Evaluation (GRADE) Working Group recommend the inclusion of stakeholder groups in the guideline development and implementation process (Akl, 2017; CIHR, 2014; NICE, 2014; WHO, 2003). The Guidelines International Network (GIN) McMaster Checklist for Guideline Development, the global standard for guideline development, identifies ‘consumer and stakeholder involvement’ as Step 6 in the guideline development process (Schünemann, 2014).

There is a growing body of research into the methods for engaging stakeholders in guideline development (Armstrong, 2017; Cluzeau, 2012), as well as approaches to measuring the impact of stakeholder engagement in guideline development (Cottrell, 2014; Ray, 2017). For example, a 2017 review of the incorporation of patients' values and preferences in guidelines included guidance documents from 56 different organisations (Selva et al., 2017). However, there remains a need for the development of comprehensive, global, evidence‐based guidance for stakeholder engagement in health guideline development that brings together the vast literature amassed to date. The Multi‐Stakeholder Engagement Consortium (MuSE Consortium) was established in 2015 to advance methods and approaches used in stakeholder engaged health research. The consortium addresses research of all kinds—including reviews and guidelines – and includes more than 80 researchers, trainees and stakeholders from Australia, Brazil, Canada, Germany, Italy, Lebanon, the Netherlands, the Philippines, Switzerland, the UK and the USA. The team membership of MuSE includes researchers, policymakers, guideline developers, research funders, clinicians, patients, patient representatives and policymakers from various organisations including the Agency for Healthcare Research and Quality (AHRQ), the Campbell Collaboration, Cochrane, GRADE Working Group, Public Health Agency of Canada, Patient‐Centered Outcomes Research Institute (PCORI), Research and Development (RAND) Corporation, the World Health Organization (WHO), Joanna Briggs Institute (JBI) and multiple universities (Petkovic, 2020). As part of the consortium's work, a working group was formed to develop global guidance on stakeholder engagement in guideline development according to the 18‐step GIN‐McMaster Checklist. The working group set out to conduct four concurrent systematic reviews to summarise the evidence on: (1) existing guidance for stakeholder engagement in guideline development, (2) barriers and facilitators to stakeholder engagement in guideline development, (3) managing conflicts of interest in stakeholder engagement in guideline development and (4) measuring the impact of stakeholder engagement in guideline development. This protocol addresses the second systematic review in the series.

### 1.2 Definitions

For the purpose of this review, we define:

- *Guidelines*as ‘systematically developed evidence‐based statements which assist providers, recipients and other stakeholders in making informed decisions about appropriate health interventions’ (WHO, 2003).
- *Stakeholders*as ‘any individual or group who is responsible for or affected by health‐ and healthcare‐related decisions that may be informed by research evidence’ (Concannon et al., 2012).
- *Engagement*as an approach to ensure the contribution of stakeholders towards the development of the guideline, completion of any of the stages of the guideline, or dissemination of the guideline and its recommendations (Frank, 2020; Pollock, 2018).
- *Levels of engagement*as the intensity of involvement in the guideline development process (Oliver, 2008; Pollock, 2019). We operationalise two levels for the purpose of this review: (1) advisory/feedback or (2) participation in decision‐making or knowledge translation.

### 1.3 Description of the phenomena of interest

Stakeholder engagement in guideline development is the approach to gather input or contribution from stakeholders ‘towards the development of a guideline, completion of any stages of a guideline, or dissemination, uptake or evaluation of a guideline and its recommendations’ (Pollock, 2018). The term ‘stakeholders’ is inclusive of multiple individuals within a single stakeholder group (such as several patients) and multiple stakeholders across several stakeholder groups (such as patients, policymakers and providers of care). Engagement refers to a meaningful and active partnership among stakeholder groups (CIHR, 2019; Staniszewska, 2017) to develop, complete or disseminate a guideline. Other terms for engagement include ‘public involvement’, ‘consumer engagement’, ‘Patient and Public Involvement (PPI)’, ‘coproduction’, ‘co‐creation’, ‘activation’ and ‘involvement’.

Stakeholder engagement in research can occur at different levels of intensity, ranging from low‐intensity communication (i.e., stakeholders are informed of research processes) up to high‐intensity coproduction (i.e., stakeholders are equal members of the research team and participate in shared decision‐making) (Concannon, 2012). From previous work (Crowe, 2017; Oliver, 2008; Pollock, 2019), we identify two levels of engagement in guideline development, namely (1) advisory/feedback, and (2) participation in decision‐making or knowledge translation. These levels were selected for our operational use, but we acknowledge that within these, there may be different levels.

**Figure 1:**
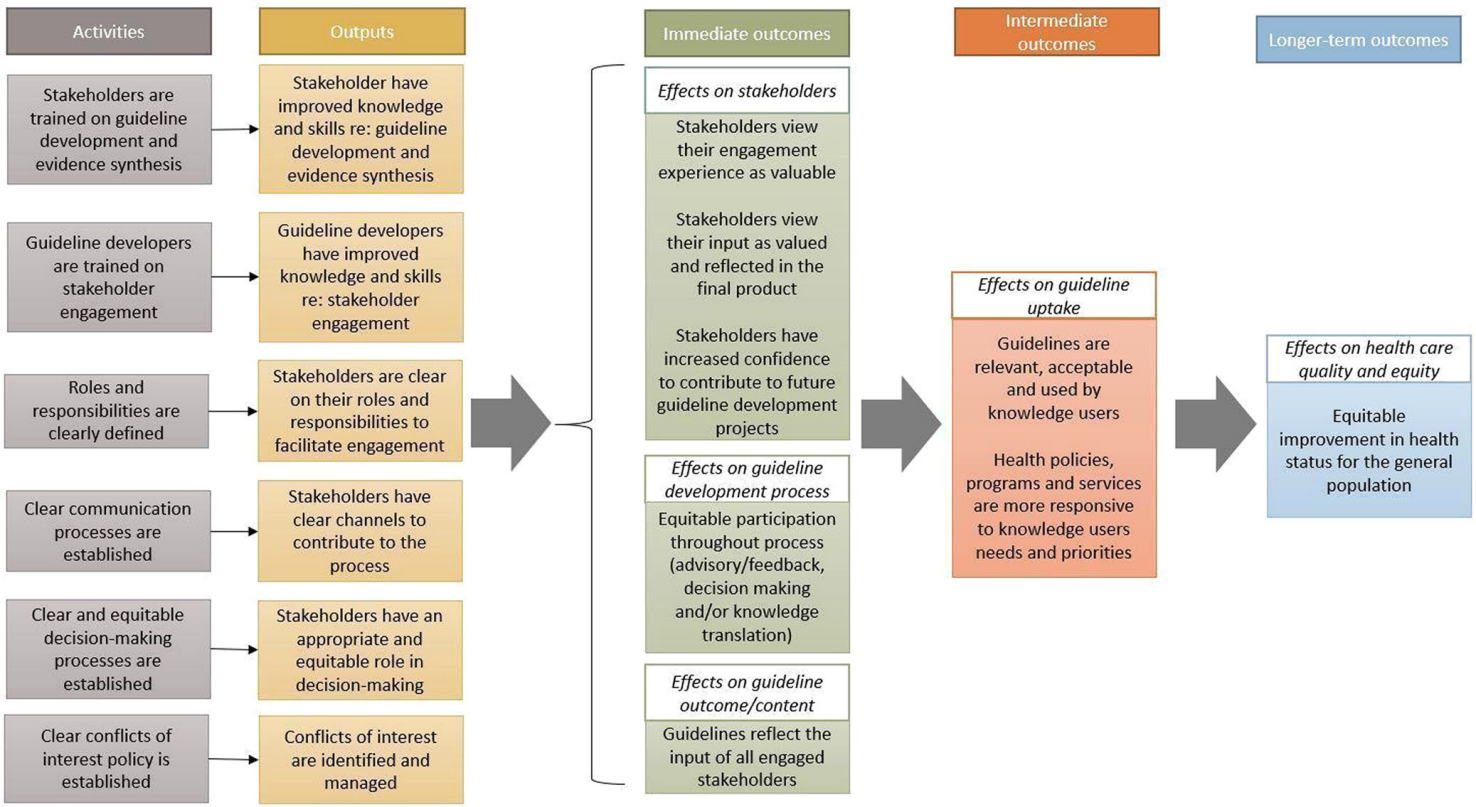
Logic model of the effects of stakeholder engagement in guideline development

There are many activities required for effective stakeholder engagement in guideline development (see Figure 1). The engagement of stakeholders from multiple disciplines or specialities is typically recommended to take account of a wide range of opinions. Indeed, the greater diversity in stakeholders' background increases the knowledge and skills available to make decisions (Oliver, 2018). However, the ability to gather and apply input from multiple stakeholders in a way that meaningfully reflects and addresses stakeholders' interests is not easily achieved, as increased diversity may also reduce group cohesiveness (Oliver, 2018). In their review of theoretical literature, Brodbeck and colleagues (Brodbeck, 2007) proposed that groups can out perform individual decision makers under conditions which promote sharing of knowledge and mutual learning. Oliver and colleagues (Oliver, 2018) built on this model, and suggested that these conditions include diverse group membership, skilled facilitation, time for knowledge sharing, following formal consensus processes, and integrating divergent perspectives. Stakeholder engagement is a complex process, and achieving these conditions requires both individual and organisational adoption. Organisational cultures and relationships act as a backdrop to these stakeholder engagement activities (Oliver, 2018). There are a multitude of individual‐ and organisational‐level factors that can affect a stakeholder's ability to engage in the guideline development process, or a guideline developer's ability to effectively engage them. Barriers to stakeholder engagement are defined as any variable, condition or factor that may impede stakeholder engagement in guideline development, while facilitators are any variable, condition or factor that may promote stakeholder engagement in guideline development. Such determinants may include knowledge, skills, time and resources, for example, and may occur across one or more steps of guideline development.

### 1.4 Why it is important to do this review

With increased recognition of the value of stakeholder engagement in guideline development, there is a need for expert opinion and evidence‐based guidance on when and how to engage stakeholders. This review of barriers and facilitators to stakeholder engagement in guideline development will help fill this gap. It will build on existing reviews of barriers and facilitators, synthesise the current evidence‐base, and examine a broad range of stakeholder groups.

To date, several systematic reviews have been conducted on the topic of stakeholder engagement in guideline development, but few are explicitly focused on barriers and facilitators to engagement. Oxman and colleagues (Oxman et al., 2006) conducted a series of reviews to advise WHO to ensure that health care recommendations are informed by the best available research evidence. Relevant reviews in this series included group composition (Fretheim, 2006a), group processes (Fretheim, 2006b) and consumer involvement (Schünemann, 2006). However, these reviews were not systematic nor exhaustive, and prioritised existing syntheses over primary empirical studies. Relatedly, Oliver and colleagues (Oliver, 2018) synthesised theoretical literature to learn about the structure, processes and environment of committees generally, which has applications for health guideline development.

Much of the existing barrier and facilitator syntheses focus specifically on patients. Légaré and colleagues (Légaré, 2011) reviewed published and unpublished literature regarding the effectiveness of patient and public engagement programs in clinical practice guidelines and described the barriers and facilitators to these programs. Barriers included recruiting challenges, lack of representation of certain patient and public groups, and participants' lack of familiarity with medical and scientific terminology. Facilitators included the provision of training and support. Similarly, Grant and colleagues (Grant, 2018) conducted a rapid review and qualitative evidence synthesis to make inferences about how feasible online methods for patient engagement would be during clinical practice guideline development. The authors highlighted important considerations for patient and caregiver participation related to skills, time and resources. Notably, both of these reviews only considered patient and public involvement and did not consider the engagement of other stakeholder groups such as healthcare providers or policymakers, for example. Recently, Selva and colleagues (Selva, 2017) reviewed guidance documents for developing clinical guidelines to assess how these documents address the incorporation of patients' views. They identified the need for additional guidance in this area.

Cluzeau and colleagues (Cluzeau, 2012; Kelson, 2012; Kunz, 2012) conducted a literature review and workshop to answer six questions related to stakeholder engagement in guideline development, including identification of the potential barriers and facilitators to integrating stakeholder involvement. They defined stakeholders as ‘all those who have a legitimate interest in a guideline. They include healthcare professionals, patients and caregivers, public and private funding bodies, managers, employers and manufacturers’. They noted that integrating different, sometimes competing, stakeholder perspectives can be challenging. Other barriers included issues with potential bias among stakeholder views, and the costly nature of the process. In contrast, they found that educating stakeholders and ensuring effective communication enabled effective stakeholder engagement.

In sum, there is a lack of systematic reviews which have synthesised barriers and facilitators to multi‐stakeholder engagement in health guideline development. The results of this review will be used to inform the development of guidance for multi‐stakeholder engagement in guideline development and implementation. This guidance will be official GRADE (Grading of Recommendations Assessment, Development and Evaluation) Working Group guidance. The GRADE system is internationally recognised as a standard for guideline development (Guyatt, 2008). The findings of this review will assist organisations who develop healthcare, public health and health policy guidelines, such as the World Health Organization, to involve multiple stakeholders in the guideline development process to ensure the development of relevant, high quality and transparent guidelines.

## 2 OBJECTIVES

The objective of this review is to identify and synthesise the existing evidence on barriers and facilitators to stakeholder engagement in health guideline development. We will address this objective through two research questions:

1. What are the barriers to multi‐stakeholder engagement in health guideline development across any of the 18 steps of the GIN‐McMaster checklist?
2. What are the facilitators to multi‐stakeholder engagement in health guideline development across any of the 18 steps of the GIN‐McMaster checklist?

## 3 METHODS

The methods for this systematic review will follow the *Cochrane Handbook for Systematic Reviews of Interventions* and the *Handbook for Synthesizing Qualitative Research*, as appropriate (Higgins, 2019; Sandelowski, 2007). These methods were developed by a multi‐stakeholder research team that engaged investigators, trainees, providers of care, policymakers and patient representatives.

### 3.1 Criteria for including and excluding studies

#### 3.1.1 Types of study designs

We will include empirical qualitative and mixed‐method primary research studies which qualitatively report on the barriers or facilitators to stakeholder engagement in health guideline development. This includes qualitative research studies using common methods such as interviews, focus groups, or surveys to collect participant experiences; case studies of existing programmes; and process evaluation studies. We will also include published guidelines which report lessons learned only if these statements are derived from empirical qualitative methods and the details of these methods are available in a published report. Mixed‐method and intervention studies (randomised or non‐randomised designs) will be eligible only if they apply qualitative methods and qualitatively report barriers and facilitators to engagement, independent of their quantitative findings.

Conference abstracts, commentaries, editorials, protocols, systematic reviews, meta‐ethnographies and other literature reviews will be excluded.

#### 3.1.2 Population of interest

The population of interest is stakeholders in health guideline development. We define stakeholders as ‘any individual or group who is responsible for or affected by health‐ and healthcare‐related decisions’ (Concannon, 2012). Building on previous work, we have identified 13 types of stakeholders whose input can enhance the relevance and uptake of guidelines (Concannon, 2012, 2019; Tugwell, 2006). We will include studies which report on the barriers or facilitators to engagement of one or several of these stakeholder groups at any level of intensity at any step of the guideline development process. For comparisons with previously published stakeholder frameworks, please see Supporting Information Appendix 7. For the purposes of this review, have grouped them as follows:

- Patients, caregivers and patient advocates
- Public
- Providers of health care
- Payers of health services
- Payers of research
- Policy makers
- Program managers
- Product makers
- Purchasers
- Principal investigators and their research teams, and
- Peer‐review editors/publishers

We recognise that within each of these categories, there is diversity and heterogeneity in types of stakeholders. For example, we recognise that patients and caregivers have differing characteristics and experiences, and their engagement brings diverse value to guideline development. The categories outlined above are for operational purposes and are based on prior research (Concannon, 2012, 2019; Tugwell, 2006). Studies which consider a single group of stakeholders or multiple groups of stakeholders will be eligible for inclusion. If a study includes multiple stakeholder groups, findings will be coded for each stakeholder group separately.

#### 3.1.3 Phenomena of interest

Eligible studies must describe stakeholder engagement at any of the following steps of the GIN‐McMaster Checklist for Guideline Development (Supporting Information Appendix 9) (Schünemann, 2014):

1. Organization, budget, planning and training
2. Priority‐setting
3. Guideline group membership
4. Establishing guideline group processes
5. Identifying target audience and topic selection
6. Consumer and stakeholder involvement
7. Conflict of interest considerations
8. Question generation
9. Considering importance of outcomes and interventions, values, preferences and utilities
10. Deciding what evidence to include and searching for evidence
11. Summarising evidence and considering additional information
12. Judging quality, strength or certainty of body of evidence
13. Developing recommendations and determining their strength
14. Wording of recommendations and of considerations about implementation, feasibility and equity
15. Reporting and peer review
16. Dissemination and implementation
17. Evaluation and use
18. Updating

This checklist was selected because it represents the global standard for guideline development. Eligible studies must report primary data in their results on barriers and facilitators to stakeholder engagement at at least one step of the checklist. Studies which report on barriers and facilitators at one step, multiple steps, or across all steps will be considered for inclusion.

#### 3.1.4 Outcomes of interest

The primary outcomes of interest are any barriers and facilitators to stakeholder engagement in health guideline development.

Barriers are defined as *any variable or condition that impedes stakeholder engagement in guideline development or implementation*.

Facilitators are defined as *any variable or condition that promotes stakeholder engagement in guideline development or implementation*.

For qualitative evidence, we will analyse first‐ and second‐order constructs related to stakeholders' perceptions and experiences of barriers and facilitators to their engagement in guideline development and implementation. These include participant quotations from interviews or focus groups, excerpts or quotations from documentary analysis, narrative descriptive summaries, author hypotheses, explanations and recommendations, themes and sub‐themes. First order‐constructs are defined as participant quotes and participant observations, while recognising that in secondary analysis these represent the participants' views as selected by the study authors in evidencing their second‐order constructs. Second‐order constructs are study authors' themes/concepts and interpretations.

#### 3.1.5 Setting

We will place no restrictions on geographic setting. Studies focused on health guideline development from any country in the world are eligible for inclusion.

#### 3.1.6 Language

No language restrictions will be applied.

#### 3.1.7 Publication date

No publication date restrictions will be applied.

### 3.2 Search methods for identification of studies

This review is part of a series of four reviews conducted by the MuSE working group on stakeholder engagement in guideline development. As such, one comprehensive search strategy was developed and peer‐reviewed in consultation with a medical librarian in accordance with PRESS guidelines (McGowan, 2016). A second medical librarian reviewed the search strategy.

#### 3.2.1 Electronic databases

We will search the following databases: MEDLINE (OVID) (Supporting Information Appendix 1), Cumulative Index to Nursing & Allied Health Literature (CINAHL; EBSCO) (Supporting Information Appendix 2), EMBASE (OVID) (Supporting Information Appendix 3), PsycInfo (OVID) (Supporting Information Appendix 4), Scopus (Supporting Information Appendix 5) and Sociological Abstracts (Supporting Information Appendix 6). Limits will not be placed on date, study design or language.

#### 3.2.2 Searching other resources

To identify grey literature, we will search the websites of agencies who actively engage stakeholder groups such as the AHRQ, Canadian Institutes of Health Research (CIHR) Strategy for Patient‐Oriented Research (SPOR), INVOLVE, the National Institute for Health and Care Excellence (NICE) and the PCORI. We will also search the websites of guideline‐producing agencies, such as the American Academy of Pediatrics, Australia's National Health Medical Research Council (NHMRC) and the WHO. We will invite members of the team to suggest grey literature sources and we plan to broaden the search by soliciting suggestions via social media, such as Twitter.

#### 3.2.3 Citations and reference lists

Backward and forward citation tracking will be performed on included articles to identify further eligible studies. We will review reference lists of relevant reviews to identify eligible primary studies for inclusion.

#### 3.2.4 Contacting experts

We will contact authors of included studies to ask for suggested studies. We will also ask our MuSE Consortium members for potentially eligible primary studies that were not identified through the database search.

#### 3.2.5 Screening of studies

All identified citations from electronic databases will be imported into Covidence software for screening and selection. Documents identified through our grey literature search will be managed and screened using an Excel spreadsheet. A two‐part study selection process will be used for all identified citations: (1) a title and abstract review and (2) full‐text review. At each stage, teams of two review authors will independently assess all potential studies in duplicate using a priori inclusion and exclusion criteria. We will resolve any disagreements through discussion or, if required, we will consult a third review author. We will produce a PRISMA flow diagram which reports the number of studies included and excluded at each stage. Reasons for exclusion will be provided for all studies assessed at full‐text.

### 3.3 Description of methods used in primary research

Relevant studies will have employed a qualitative or mixed‐method design. For example:

- Brouwers et al. (2017) (mixed‐methods; Brouwers, 2017). Cancer patients, survivors, family members and caregivers were recruited to explore optimal approaches to patient and caregiver engagement in the development of cancer practice guidelines. Participants attended a workshop, completed a survey, or participated in a telephone interview. Quantitative data were analysed using Microsoft Excel, and qualitative data were analysed using thematic analysis. Authors report on barriers and facilitators to engaging in guideline development.
- Atkins et al. (2013) (qualitative; Atkins, 2013). Members of three National Institute of Health and Clinical Excellence (NICE) advisory groups on acute physical, mental and public health were interviewed to investigate how they conceptualise evidence and experience the process. Participants were interviewed at the beginning and end of the life of the group. Interview transcripts were analysed using thematic and content analysis to identify main themes.

### 3.4 Criteria for determination of independent findings

#### 3.4.1 Multiple reports of the same study

If a guideline development process is evaluated by more than one publication and is published in multiple reports, we will only include the most recent report or any additional report with unique outcome assessments (e.g., secondary analysis reports). If several reports of the same guideline process are identified and all publications report on different outcomes, each individual report must meet eligibility criteria for inclusion. Eligible reports of the same study will be analysed as a single study. Information on study sample sizes, guideline details, grant numbers and so on will be used to identify multiple reports from single studies and multiple studies in single reports. The authors of the reports will be contacted if it is unclear whether reports and studies provide independent findings.

#### 3.4.2 Multiple studies in single reports

If more than one study is described in a single report, each study within the report will be coded separately.

#### 3.4.3 Multiple studies involving the same guideline development team

Reports published by the same guideline development team are likely to follow similar stakeholder engagement practices, and so multiple reports published by a single guideline team will be aggregated.

### 3.5 Details of study coding categories

Data will be extracted by two review authors independently and in duplicate according to a standardised data extraction form (see Supporting Information Appendix 8). Disagreements will be discussed and resolved by a third reviewer. The data extraction form will be pilot tested by two review authors and coding categories will be clarified iteratively, as needed. Data will be extracted on basic study characteristics and methods including:

- Study objective
- Study setting
- Type of guideline
- Population and participant characteristics, including categorisation according to stakeholder group and equity‐relevant characteristics according to PROGRESS‐Plus (O'Neill, 2014).
- Recruitment methods
- Data collection methods
- Analysis methods

In addition, we will extract information on study:

- Limitations
- Recommendations
- Conclusions
- Funding
- Conflict of Interest

For qualitative outcomes, we will extract data using codes adopted from the Theoretical Domains Framework (Atkins, 2017). We will extract stakeholders' perceptions and experiences regarding the barriers and facilitators to their engagement in health guideline development from the results and discussion sections, including participant quotations from interviews or focus groups, excerpts or quotations from documentary analysis, narrative descriptive summaries, author hypotheses, explanations and recommendations, themes and sub‐themes.

Discrepancies in the data extraction process will be resolved by a third reviewer.

#### 3.5.1 Dealing with missing data

Missing data is an inherent component of the qualitative research process. Since meanings and interpretations are the outputs of qualitative studies, data are always missing because there are some questions that respondents do not want to answer, others they circumvent, and aspects of their experiences which may not be reported (Singh, 2013). In such situations, the silence on these issues in itself becomes powerful data. Our findings will be mapped against the GIN‐McMaster Checklist and we will report which steps of the checklist do have supporting qualitative evidence and those which do not, thereby highlighting gaps in evidence and opportunities for new qualitative inquiry.

### 3.6 Critical appraisal

Two review authors will assess the methodological rigour of all included studies independently and in duplicate. We will assess the quality of qualitative studies and the qualitative components of mixed method studies using the Critical Appraisal Skills Programme (CASP) qualitative appraisal research tool (Singh, 2013). Discrepancies in the critical appraisal process will be resolved by a third reviewer.

### 3.7 Treatment of qualitative research

#### 3.7.1 Identification of key findings

Our included studies will employ a qualitative approach to inquiry. We will use the ‘best fit’ framework method as a systematic and flexible approach to analysing the qualitative data (Booth, 2015; Carroll, 2013; Gale, 2013). Framework‐based synthesis, using the ‘best fit’ method, is a highly pragmatic and useful strategy for analysing a range of questions (Dixon‐Woods, 2011) and is supported by guideline development literature (Flemming, 2019). Framework analysis is a five‐stage process that includes familiarisation with the data, identifying a thematic framework, indexing (applying the framework), charting and mapping and interpretation (Spencer & Ritchie, 1994).

Implementing new or changing existing stakeholder engagement practices, in organisations who develop guidelines, requires a change in individual and collective behaviour. Changing behaviour requires an understanding of the influences on behaviour in the context in which they occur. We selected the Theoretical Domains Framework (TDF) for extracting and analysing our barriers and facilitator data (Atkins, 2017). Developed through a multidisciplinary consensus approach and subsequent validation, TDF consolidates overlapping behavioural theories into 14 domains encompassing 84 theoretical constructs, and provides a theoretical lens through which to view the cognitive, affective, social and environmental influences on behaviour and implementation.

Data will be analysed deductively, using the TDF domains to generate a framework of preliminary themes. Whilst the domains are purposively designed to be broad groupings of the possible factors to influence behaviour, the intent is to explore the important domains in further detail. Data will be coded independently by two reviewers. Disagreements will be resolved by discussion. The entire review team will be involved in developing key findings.

#### 3.7.2 Mapping of key findings

All identified key findings will be mapped to the 18 topics (146 steps) of the guideline development process outlined by the GIN‐McMaster checklist (Shunemann 2014). We will present this information as a matrix indicating the barriers and facilitators that exists for each stakeholder group and for each step of guideline development. The findings of this mapping exercise will be combined with the results of the other reviews in this series (Khabsa, 2022; Petkovic, 2022) to develop international guidance on stakeholder engagement in guideline development.

### 3.8 Certainty of evidence

We will use the Confidence in the Evidence from Reviews of Qualitative research (CERQual) tool to assess the confidence of our findings. This tool is a method for assessing the strength of qualitative review evidence, similar to how the GRADE approach assesses the strength of quantitative evidence (Lewin et al., 2018). CERQual bases the evaluation on four criteria: (a) methodological limitations of included studies supporting a review finding, (b) the relevance of included studies to the review question, (c) the coherence of the review finding and (d) the adequacy of the data contributing to a review finding. Final CERQual assessments will be presented as a CERQual Evidence Profile.

## CONTRIBUTIONS OF AUTHORS

Conceiving the review: PT, VW, TC, JP
Designing the review: JP, AR, VW, PT, TC
Coordinating the review: AR, OM, JP
Writing the protocol: AR, OM, JP, PA, JK, LL
Providing general advice on the review: EAA, MS, RM, VW, LD, NS, PC, SG
Securing funding for the review: PT, VW, JP

## DECLARATIONS OF INTEREST

None.

## PUBLISHED NOTES

None.

## SOURCES OF SUPPORT

Internal sources

• No sources of support provided

External sources

• Canadian Institutes for Health Research, Canada

## Supporting information

Supporting information.
