# CORRIGENDUM

**DOI:** 10.1002/cl2.1248

**Published:** 2022-06-01

The authors of Magwood et al. (2022) have supplied the following correction to their article.

One of the coauthor names was incorrectly spelled in the article as ‘Tom Concannon’. The coauthor's name is being corrected to ‘Thomas W. Concannon’.

The original version is also corrected and can be found on the below link: https://doi.org/10.1002/cl2.1237. The authors apologise for the error.
