# Supplementary material for: PROTOCOL: Barriers and facilitators to stakeholder engagement in health guideline development: A qualitative evidence synthesis
Source: Campbell Syst Rev. 2022 Apr 25;18(2):e1237. doi: 10.1002/cl2.1237 (PMC9038083; doi:10.1002/cl2.1237)
Supplement: Supplementary file 1 — Supporting information. [file CL2-18-e1237-s001.docx]

# Appendices

## 1 Medline Search Strategy

1 Stakeholder Participation/

2 patient participation/

3 consumer participation/

4 Community-Based Participatory Research/

5 (coproduction or co-production).ti,ab,kf.

6 ((stakeholder* or advisor* or reference* or expert* or consultation* or steering) adj2 (group* or panel*)).ti,ab,kf.

7 ((stakeholder* or patient* or consumer* or public or caregiver* or care-giver* or communit* or citizen* or user* or service-user* or end-user* or clinician* or doctor* or physician* or nurse* or policymaker* or policy-maker* or funder* or indust* or pharmaceutical) adj2 (engag* or involv* or input or participat* or collaborat*)).ti,ab,kf.

8 or/1-7

9 practice guidelines as topic/

10 Guideline*.ti.

11 Guidance.ti,kf.

12 Clinical guideline*.ti,ab,kf.

13 Clinical Practice Guideline*.ti,ab,kf.

14 or/9-13

15 8 and 14 (6569)

## 2 CINAHL Search Strategy

| **#** | **Query** | **Limiters/Expanders** | **Last Run Via** |
| --- | --- | --- | --- |
| S11 | S5 AND S10 | Search modes - Boolean/Phrase | Interface - EBSCOhost Research Databases Search Screen - Advanced Search Database - CINAHL Complete |
| S10 | S6 OR S7 OR S8 OR S9 | Search modes - Boolean/Phrase | Interface - EBSCOhost Research Databases Search Screen - Advanced Search Database - CINAHL Complete |
| S9 | (clinical OR practice) N1 guideline* | Search modes - Boolean/Phrase | Interface - EBSCOhost Research Databases Search Screen - Advanced Search Database - CINAHL Complete |
| S8 | TI Guidance OR SU Guidance | Search modes - Boolean/Phrase | Interface - EBSCOhost Research Databases Search Screen - Advanced Search Database - CINAHL Complete |
| S7 | TI Guideline* | Search modes - Boolean/Phrase | Interface - EBSCOhost Research Databases Search Screen - Advanced Search Database - CINAHL Complete |
| S6 | MH Practice Guidelines | Search modes - Boolean/Phrase | Interface - EBSCOhost Research Databases Search Screen - Advanced Search Database - CINAHL Complete |
| S5 | S1 OR S2 OR S3 OR S4 | Search modes - Boolean/Phrase | Interface - EBSCOhost Research Databases Search Screen - Advanced Search Database - CINAHL Complete |
| S4 | (stakeholder* OR patient* OR consumer* OR public OR caregiver* OR communit* OR citizen* OR user* OR service-user* OR end-user* OR clinician* OR doctor* OR physician* OR nurse* OR policymaker* OR funder* OR industry OR pharmaceutical) N2 (engag* OR involv* OR input OR participat*) | Search modes - Boolean/Phrase | Interface - EBSCOhost Research Databases Search Screen - Advanced Search Database - CINAHL Complete |
| S3 | (stakeholder* OR advisor* OR reference* OR expert* OR consultation OR steering) N2 (group* OR panel) | Search modes - Boolean/Phrase | Interface - EBSCOhost Research Databases Search Screen - Advanced Search Database - CINAHL Complete |
| S2 | coproduction OR co-production | Search modes - Boolean/Phrase | Interface - EBSCOhost Research Databases Search Screen - Advanced Search Database - CINAHL Complete |
| S1 | MH Consumer Participation | Search modes - Boolean/Phrase | Interface - EBSCOhost Research Databases Search Screen - Advanced Search Database - CINAHL Complete |

## 3 Embase Search Strategy

1 Stakeholder Participation/

2 patient participation/

3 consumer participation/

4 Community-Based Participatory Research/

5 (coproduction or co-production).ti,ab,kf.

6 ((stakeholder* or advisor* or reference* or expert* or consultation or steering) adj2 (group* or panel)).ti,ab,kf.

7 ((stakeholder* or patient* or consumer* or public or communit* or citizen* or user* or service-user* or end-user* or clinician* or doctor* or physician* or nurse* or policymaker* or funder* or industry or pharmaceutical) adj2 (engag* or involv* or input or participat*)).ti,ab,kf.

8 or/1-7

9 practice guidelines as topic/

10 Guideline*.ti.

11 Clinical guideline*.ti,ab,kf.

12 Clinical Practice Guideline*.ti,ab,kf.

13 or/9-12

14 8 and 13

## 4 PsycINFO Search Strategy

1 Client Participation/

2 Stakeholder/ and Involvement/

3 Stakeholder/ and Participation/

4 coproduction.ti,ab.

5 co-production.ti,ab.

6 (stakeholder* adj2 group*).mp. [mp=title, abstract, heading word, table of contents, key concepts, original title, tests & measures]

7 (stakeholder* adj2 panel).mp. [mp=title, abstract, heading word, table of contents, key concepts, original title, tests & measures]

8 (advisor* adj2 group*).mp. [mp=title, abstract, heading word, table of contents, key concepts, original title, tests & measures]

9 (advisor* adj2 panel).mp. [mp=title, abstract, heading word, table of contents, key concepts, original title, tests & measures]

10 (reference* adj2 group*).mp. [mp=title, abstract, heading word, table of contents, key concepts, original title, tests & measures]

11 (reference* adj2 panel).mp. [mp=title, abstract, heading word, table of contents, key concepts, original title, tests & measures]

12 (expert* adj2 group*).mp. [mp=title, abstract, heading word, table of contents, key concepts, original title, tests & measures]

13 (expert* adj2 panel).mp. [mp=title, abstract, heading word, table of contents, key concepts, original title, tests & measures]

14 (consultation adj2 group*).mp. [mp=title, abstract, heading word, table of contents, key concepts, original title, tests & measures]

15 (consultation adj2 panel).mp. [mp=title, abstract, heading word, table of contents, key concepts, original title, tests & measures]

16 (steering adj2 group*).mp. [mp=title, abstract, heading word, table of contents, key concepts, original title, tests & measures]

17 (steering adj2 panel).mp. [mp=title, abstract, heading word, table of contents, key concepts, original title, tests & measures]

18 (stakeholder* adj2 engag*).mp. [mp=title, abstract, heading word, table of contents, key concepts, original title, tests & measures]

19 (stakeholder* adj2 involv*).mp. [mp=title, abstract, heading word, table of contents, key concepts, original title, tests & measures]

20 (stakeholder* adj2 input).mp. [mp=title, abstract, heading word, table of contents, key concepts, original title, tests & measures]

21 (stakeholder* adj2 participat*).mp. [mp=title, abstract, heading word, table of contents, key concepts, original title, tests & measures]

22 (patient* adj2 engag*).mp. [mp=title, abstract, heading word, table of contents, key concepts, original title, tests & measures]

23 (patient* adj2 involv*).mp. [mp=title, abstract, heading word, table of contents, key concepts, original title, tests & measures]

24 (patient* adj2 input).mp. [mp=title, abstract, heading word, table of contents, key concepts, original title, tests & measures]

25 (patient* adj2 participat*).mp. [mp=title, abstract, heading word, table of contents, key concepts, original title, tests & measures]

26 (consumer* adj2 engag*).mp. [mp=title, abstract, heading word, table of contents, key concepts, original title, tests & measures]

27 (consumer* adj2 involv*).mp. [mp=title, abstract, heading word, table of contents, key concepts, original title, tests & measures]

28 (consumer* adj2 input).mp. [mp=title, abstract, heading word, table of contents, key concepts, original title, tests & measures]

29 (consumer* adj2 participat*).mp. [mp=title, abstract, heading word, table of contents, key concepts, original title, tests & measures]

30 (public adj2 engag*).mp. [mp=title, abstract, heading word, table of contents, key concepts, original title, tests & measures]

31 (public adj2 involv*).mp. [mp=title, abstract, heading word, table of contents, key concepts, original title, tests & measures]

32 (public adj2 input).mp. [mp=title, abstract, heading word, table of contents, key concepts, original title, tests & measures]

33 (public adj2 participat*).mp. [mp=title, abstract, heading word, table of contents, key concepts, original title, tests & measures]

34 (caregiver* adj2 engag*).mp. [mp=title, abstract, heading word, table of contents, key concepts, original title, tests & measures]

35 (caregiver* adj2 involv*).mp. [mp=title, abstract, heading word, table of contents, key concepts, original title, tests & measures]

36 (caregiver* adj2 input).mp. [mp=title, abstract, heading word, table of contents, key concepts, original title, tests & measures]

37 (caregiver* adj2 participat*).mp. [mp=title, abstract, heading word, table of contents, key concepts, original title, tests & measures]

38 (communit* adj2 engag*).mp. [mp=title, abstract, heading word, table of contents, key concepts, original title, tests & measures]

39 (communit* adj2 involv*).mp. [mp=title, abstract, heading word, table of contents, key concepts, original title, tests & measures]

40 (communit* adj2 input).mp. [mp=title, abstract, heading word, table of contents, key concepts, original title, tests & measures]

41 (communit* adj2 participat*).mp. [mp=title, abstract, heading word, table of contents, key concepts, original title, tests & measures]

42 (citizen* adj2 engag*).mp. [mp=title, abstract, heading word, table of contents, key concepts, original title, tests & measures]

43 (citizen* adj2 involv*).mp. [mp=title, abstract, heading word, table of contents, key concepts, original title, tests & measures]

44 (citizen* adj2 input).mp. [mp=title, abstract, heading word, table of contents, key concepts, original title, tests & measures]

45 (citizen* adj2 participat*).mp. [mp=title, abstract, heading word, table of contents, key concepts, original title, tests & measures]

46 (user* adj2 engag*).mp. [mp=title, abstract, heading word, table of contents, key concepts, original title, tests & measures]

47 (user* adj2 involv*).mp. [mp=title, abstract, heading word, table of contents, key concepts, original title, tests & measures]

48 (user* adj2 input).mp. [mp=title, abstract, heading word, table of contents, key concepts, original title, tests & measures]

49 (user* adj2 participat*).mp. [mp=title, abstract, heading word, table of contents, key concepts, original title, tests & measures]

50 (service-user* adj2 engag*).mp. [mp=title, abstract, heading word, table of contents, key concepts, original title, tests & measures]

51 (service-user* adj2 involv*).mp. [mp=title, abstract, heading word, table of contents, key concepts, original title, tests & measures]

52 (service-user* adj2 input).mp. [mp=title, abstract, heading word, table of contents, key concepts, original title, tests & measures]

53 (service-user* adj2 participat*).mp. [mp=title, abstract, heading word, table of contents, key concepts, original title, tests & measures]

54 (end-user* adj2 engag*).mp. [mp=title, abstract, heading word, table of contents, key concepts, original title, tests & measures]

55 (end-user* adj2 involv*).mp. [mp=title, abstract, heading word, table of contents, key concepts, original title, tests & measures]

56 (end-user* adj2 input).mp. [mp=title, abstract, heading word, table of contents, key concepts, original title, tests & measures]

57 (end-user* adj2 participat*).mp. [mp=title, abstract, heading word, table of contents, key concepts, original title, tests & measures]

58 (clinician* adj2 engag*).mp. [mp=title, abstract, heading word, table of contents, key concepts, original title, tests & measures]

59 (clinician* adj2 involv*).mp. [mp=title, abstract, heading word, table of contents, key concepts, original title, tests & measures]

60 (clinician* adj2 input).mp. [mp=title, abstract, heading word, table of contents, key concepts, original title, tests & measures]

61 (clinician* adj2 participat*).mp. [mp=title, abstract, heading word, table of contents, key concepts, original title, tests & measures]

62 (doctor* adj2 engag*).mp. [mp=title, abstract, heading word, table of contents, key concepts, original title, tests & measures]

63 (doctor* adj2 involv*).mp. [mp=title, abstract, heading word, table of contents, key concepts, original title, tests & measures]

64 (doctor* adj2 input).mp. [mp=title, abstract, heading word, table of contents, key concepts, original title, tests & measures]

65 (doctor* adj2 participat*).mp. [mp=title, abstract, heading word, table of contents, key concepts, original title, tests & measures]

66 (physician* adj2 engag*).mp. [mp=title, abstract, heading word, table of contents, key concepts, original title, tests & measures]

67 (physician* adj2 involv*).mp. [mp=title, abstract, heading word, table of contents, key concepts, original title, tests & measures]

68 (physician* adj2 input).mp. [mp=title, abstract, heading word, table of contents, key concepts, original title, tests & measures]

69 (physician* adj2 participat*).mp. [mp=title, abstract, heading word, table of contents, key concepts, original title, tests & measures]

70 (nurse* adj2 engag*).mp. [mp=title, abstract, heading word, table of contents, key concepts, original title, tests & measures]

71 (nurse* adj2 involv*).mp. [mp=title, abstract, heading word, table of contents, key concepts, original title, tests & measures]

72 (nurse* adj2 input).mp. [mp=title, abstract, heading word, table of contents, key concepts, original title, tests & measures]

73 (nurse* adj2 participat*).mp. [mp=title, abstract, heading word, table of contents, key concepts, original title, tests & measures]

74 (policymaker* adj2 engag*).mp. [mp=title, abstract, heading word, table of contents, key concepts, original title, tests & measures]

75 (policymaker* adj2 involv*).mp. [mp=title, abstract, heading word, table of contents, key concepts, original title, tests & measures]

76 (policymaker* adj2 input).mp. [mp=title, abstract, heading word, table of contents, key concepts, original title, tests & measures]

77 (policymaker* adj2 participat*).mp. [mp=title, abstract, heading word, table of contents, key concepts, original title, tests & measures]

78 (funder* adj2 engag*).mp. [mp=title, abstract, heading word, table of contents, key concepts, original title, tests & measures]

79 (funder* adj2 involv*).mp. [mp=title, abstract, heading word, table of contents, key concepts, original title, tests & measures]

80 (funder* adj2 input).mp. [mp=title, abstract, heading word, table of contents, key concepts, original title, tests & measures]

81 (funder* adj2 participat*).mp. [mp=title, abstract, heading word, table of contents, key concepts, original title, tests & measures]

82 (industry adj2 engag*).mp. [mp=title, abstract, heading word, table of contents, key concepts, original title, tests & measures]

83 (industry adj2 involv*).mp. [mp=title, abstract, heading word, table of contents, key concepts, original title, tests & measures]

84 (industry adj2 input).mp. [mp=title, abstract, heading word, table of contents, key concepts, original title, tests & measures]

85 (industry adj2 participat*).mp. [mp=title, abstract, heading word, table of contents, key concepts, original title, tests & measures]

86 (pharmaceutical adj2 engag*).mp. [mp=title, abstract, heading word, table of contents, key concepts, original title, tests & measures]

87 (pharmaceutical adj2 involv*).mp. [mp=title, abstract, heading word, table of contents, key concepts, original title, tests & measures]

88 (pharmaceutical adj2 input).mp. [mp=title, abstract, heading word, table of contents, key concepts, original title, tests & measures]

89 (pharmaceutical adj2 participat*).mp. [mp=title, abstract, heading word, table of contents, key concepts, original title, tests & measures]

90 1 or 2 or 3 or 4 or 5 or 6 or 7 or 8 or 9 or 10 or 11 or 12 or 13 or 14 or 15 or 16 or 17 or 18 or 19 or 20 or 21 or 22 or 23 or 24 or 25 or 26 or 27 or 28 or 29 or 30 or 31 or 32 or 33 or 34 or 35 or 36 or 37 or 38 or 39 or 40 or 41 or 42 or 43 or 44 or 45 or 46 or 47 or 48 or 49 or 50 or 51 or 52 or 53 or 54 or 55 or 56 or 57 or 58 or 59 or 60 or 61 or 62 or 63 or 64 or 65 or 66 or 67 or 68 or 69 or 70 or 71 or 72 or 73 or 74 or 75 or 76 or 77 or 78 or 79 or 80 or 81 or 82 or 83 or 84 or 85 or 86 or 87 or 88 or 89

91 Treatment Guidelines/

92 Guideline*.ti.

93 Guidance.ti.

94 guidance.id.

95 (clinical adj1 guideline*).mp. [mp=title, abstract, heading word, table of contents, key concepts, original title, tests & measures]

96 (practice adj1 guideline*).mp. [mp=title, abstract, heading word, table of contents, key concepts, original title, tests & measures]

97 91 or 92 or 93 or 94 or 95 or 96

98 90 and 97

## 5 Scopus Search Strategy

( ( TITLE-ABS-KEY ( coproduction OR co-production ) ) OR ( TITLE-ABS-KEY ( ( stakeholder* OR advisor* OR reference* OR expert* OR consultation* OR steering ) W/2 ( group* OR panel* ) ) ) OR ( TITLE-ABS-KEY ( ( stakeholder* OR patient* OR consumer* OR public OR caregiver* OR care-giver* OR communit* OR citizen* OR user* OR service-user* OR end-user* OR clinician* OR doctor* OR physician* OR nurse* OR policymaker* OR policy-maker* OR funder* OR industr* OR pharmaceutical ) W/2 ( engag* OR involv* OR input OR participat* OR collaborat* ) ) ) ) AND ( ( TITLE ( guideline* ) ) OR ( TITLE ( guidance ) ) OR ( KEY ( guidance ) ) OR ( TITLE-ABS-KEY ( "clinical guideline*" OR "clinical practice guideline*" ) ) )

## 6 Sociological Abstracts Search Strategy

Searched for: (((MAINSUBJECT.EXACT ("Audience participation") OR MAINSUBJECT.EXACT("Participation") OR MAINSUBJECT.EXACT("Stakeholders") OR MAINSUBJECT.EXACT("Community") OR MAINSUBJECT.EXACT("Citizen participation") OR MAINSUBJECT.EXACT("Consumers")) OR noft((stakeholder* OR advisor* OR reference* OR expert* OR consultation* OR steering) NEAR/2 (group* OR panel*)) OR noft((stakeholder* OR patient* OR consumer* OR public OR caregiver* OR care-giver* OR communit* OR citizen* OR user* OR service-user* OR end-user* OR clinician* OR doctor* OR physician* OR nurse* OR policymaker* OR policy-maker* OR funder* OR indust* OR pharmaceutical) NEAR/2 (engag* OR involv* OR input OR participat* OR collaborat*))) AND (MAINSUBJECT.EXACT("Guidelines") OR ti(Guideline*) OR noft(Guidance*) OR noft(Clinical NEAR/3 guideline*))) AND stype.exact("Scholarly Journals")

## 7 Stakeholder Models

| **Tufts-RAND 7Ps Mode** | | **PCORI Model** | | **CMTP Model** | | **Cochrane Model** | | **Ottawa CGH 6Ps Model** | **MuSE Application for Systematic Review and Clinical Guidelines Developers** | |
| --- | --- | --- | --- | --- | --- | --- | --- | --- | --- | --- |
| **Type** | **Description** | **Type** | **Description** | **Type** | **Description** | **Type** | **Description** | **Type** | **Type** | **Description** |
| **Patients and the Public** | Current and potential consumers of patient-centered health care and population-focused public health, their caregivers, families and patient and consumer advocacy organizations | **Patients and consumers** | Persons or organizations that represent the patient or consumer perspective generally, or within specific disease states, such as individuals with particular conditions, caregivers, patient advocates and advocacy organizations | **Patients** | Persons with current or past experience of illness or injury, family members or other unpaid caregivers of patients, or members of advocacy organizations that represent patients or caregivers | **Consumers and the public** | Those seeking health care, their families and careers, and the publ**i**c | **Patient** | **Patients** | Persons who have experience with the condition/disease of interest |
|  |  |  |  |  |  |  |  |  | **Caregivers** | Family members and others who provide unpaid care to a patient |
|  |  |  |  |  |  |  |  |  | **Patient Advocates** | Persons and organizations that advocate for patients and caregivers, usually within one specific clinical condition or population |
|  |  |  |  |  |  |  |  | **Public** | **Public** | The general population within a defined geographic area, excluding patients, caregivers, and health professionals living or working with the condition of interest. |
| **Providers** | Individuals (e.g. nurses, physicians, mental health counselors, pharmacists, and other providers of care and support services) and organizations (e.g. hospitals, clinics, community health centers, community-based organizations, pharmacies, EMS agencies, skilled nursing facilities, schools) that provide care to patients and population | **Clinicians** | Individuals who provide healthcare services, such as physicians, nurses, pharmacists, nurse practitioners, physician assistants and mental health providers | **Clinicians** | Providers of health care in a clinical setting, including physicians, nurses, physician assistants, rehabilitative professionals, pharmacists, mental healthcare providers, complementary and alternative healthcare providers, and professional societies serving clinicians | **Practitioners** | [Practitioners] of health care including clinicians and public health practitioners | **Practitioner** | **Providers** | Persons--and their professional associations--who provide health care in a professional capacity |
|  |  | **Healthcare Providers** | Institutions that deliver healthcare services, such as hospitals, nursing homes, outpatient clinics, clinical laboratories and accountable healthcare organizations | **Hospitals and Healthcare Systems** | Organizations where care is delivered, including public and private hospitals and health systems, urgent care centers, retail health clinics, and community health centers, and organizations representing these facilities | **Policy Makers & Health Care Managers** | [Individuals and entities] making decisions about health policy within all levels of management |  | **Program Managers** | Entities that deliver health care to patients |
| **Payers** | Insurers, Medicare and Medicaid, state insurance exchanges, individuals with deductibles, and others responsible for reimbursement for interventions and episodes of care | **Payers and Purchasers** | Organizations that pay for healthcare goods and services, such as public and private insurers, health plans and employers | **Payers** | Those who function as financial intermediaries in the health system, including private insurers and public insurers, and organizations representing insurers, such as America’s Health Insurance Plans |  |  |  | **Payers of Health Services** | Individuals, organizations and entities that pay for health services |
| **Purchases** | Employers, the self-insured, government and other entities responsible for underwriting the costs of health care |  |  | **Purchasers** | Those who purchase health benefits for employees and their dependents, including individual businesses as well as local, state, regional, and national business groups, coalitions that represent businesses, and health coalitions |  |  |  | **Purchasers** | Organizations and entities – such as employers and government -- that purchase health benefits and insurance for individuals and families. |
| **Product makers** | Drug and device manufacturers | **Life Sciences Industry** | Entities that develop and market medical technologies, such as pharmaceutical, medical device, diagnostic, biotechnology companies and organizations that represent life science company interests | **Industry** | Companies that design, invest in, or manufacture diagnostics, devices, pharmaceuticals, electronic records systems, and mobile apps, and organizations representing the life sciences industry, such as the Advanced Medical Technologies Association |  |  | **Private Sector** | **Product Makers** | Organizations and entities that manufacture diagnostics, medical devices, pharmaceuticals, bio-technology, electronic records systems, mobile apps, and other products that are used in the delivery or consumption of health services. |
| **Policy Makers** | The White House, Department of Health and Human Services, Congress, states, professional associations, intermediaries, and other policy-making entities | **Policy makers and Regulators** | Individuals and organizations that create, monitor and oversee policies or regulations of healthcare-related issues, such as federal, state and local government agencies, medical and professional organizations and clinical guideline developers | **Policy makers** | Those who help craft public policy at any level of government, including federal, state, and local government officials; federal, state, and local units of government; and organizations that represent policy makers | **Policy Makers & Health Care Managers** | [Individuals and entities] making decisions about health policy within all levels of management | **Policy Maker** | **Policy Makers** | Individuals, organizations and entities that craft public or private policy (on health) at any level of government (e.g. politicians, national, provincial, state, or local, scientific advisors) |
|  |  | **Training Institutions** | Those that deliver health professional education include public and private universities and colleges, individuals affiliated with the delivery or administration of health professional education, and trade or professional associations representing these institutions, organizations, and individuals |  |  |  |  |  |  |  |
| **Principal Investigators** | Other researchers and their funders | **Researchers** | Individuals and their related organizations that develop scientific and clinical evidence, such as clinical researchers, health services researchers, social scientists and basic scientists | **Researchers** | Those who conduct clinical research, including investigators or funders of research and organizations or associations representing the research community | **Researchers & Research Funders** | [Individuals and entities] who need information regarding important gaps in the evidence |  | **Principal Investigators** | Individuals, organizations, and associations that conduct or advocate health research. |
|  |  | **Research Funders** | Entities that provide monetary support for research efforts, such as government, foundations and for-profit organizations |  |  |  |  |  | **Payers of Health Research** | Public and private organizations that fund health research. |
|  |  |  |  |  |  |  |  | **Press** | **Peer- review editors** | Individuals who manage peer review or edit peer reviewed research. |

## 8 Data extraction codebook

| Methodology | | | | | | | | Results | | | Discussion/Conclusion | | | | | |
| --- | --- | --- | --- | --- | --- | --- | --- | --- | --- | --- | --- | --- | --- | --- | --- | --- |
| Study design | Study Objective | Study setting | Type of guideline | Participant eligibility criteria | Sampling and Recruitment methods | Data collection methods | Data analysis methods | Participant characteristics | This article discusses barriers/facilitators to engaging which of the following stakeholders? (select all that apply) | | Study Limitations | Recommendations | Conclusions | Funding | Conflict of Interest | Miscellaneous |
| TDF Domain | | | | | | | | | | | | | | |  |  |
| Knowledge: An awareness of the existence of something | Skills: An ability or proficiency acquired through practice | Social/Professional Role and Identity: A coherent set of behaviours and displayed personal qualities of an individual in a social or work setting | Beliefs about Capabilities: Acceptance of the truth, reality, or validity about an ability, talent, or facility that a person can put to constructive use | Optimism: The confidence that things will happen for the best or that desired goals will be attained | Beliefs about Consequences: Acceptance of the truth, reality, or validity about outcomes of a behaviour in a given situation | Reinforcement: Increasing the probability of a response by arranging a dependent relationship, or contingency, between the response and a given stimulus | Intentions: A conscious decision to perform a behaviour or a resolve to act in a certain way | Goals: Mental representations of outcomes or end states that an individual wants to achieve | Memory, Attention and Decision Processes: The ability to retain information, focus selectively on aspects of the environment and choose between two or more alternatives | Environmental Context and Resources: Any circumstance of a person's situation or environment that discourages or encourages the development of skills and abilities, independence, social competence, and adaptive behaviour | Social influences: Those interpersonal processes that can cause individuals to change their thoughts, feelings, or behaviours | Emotion: A complex reaction pattern, involving experiential, behavioural, and physiological elements, by which the individual attempts to deal with a personally significant matter or event | Behavioural Regulation: Anything aimed at managing or changing objectively observed or measured actions  Ex: Self-monitoring; Breaking habit; Action planning | Other (anything that did not fit in previous domains) |  |  |

## 9 GIN-McMaster Checklist

| **Guideline Development Steps** |
| --- |
| ***1. Organization, Budget, Planning and Training*** |
| 1. Establish the structure of the guideline development group and determine the roles, tasks, and relationships among the various groups to be involved (e.g. oversight committee/body to direct guideline topic selection and group membership, working group consisting of experts and methodologists to synthesize evidence, a secretariat to provide administrative support, guideline panel to develop recommendations, and stakeholders and consumers for consultation). (see Topics 3, 4 & 6) |
| 2. Perform a thorough assessment of the proposed guideline development project with respect to financial and feasibility issues concerning the guideline development group (e.g. availability of resources to complete the project, expected commitment from guideline panel and staff, etc.). |
| 3. Obtain organizational approval to proceed with the guideline project. |
| 4. Prepare a budget for the development of the guideline, outlining the estimated costs for each step (e.g. working group and staff remuneration, outsourcing of certain tasks to outside organizations or groups, travel expenses, publication and dissemination expenses, etc.). |
| 5. Determine whether guideline panel members will be provided any payment or reimbursement for their time or will work as volunteers. |
| 6. Obtain or secure funding for the development of the guideline, with attention to conflict of interest considerations. (see Topic 7) |
| 7. Outline and arrange the administrative support that will be required to facilitate the guideline development process (e.g. a secretariat of the working group to organize and obtain declaration of interests, arrange group meetings, etc.). |
| 8. Plan and prepare for training and support that will be required for those involved in the guideline development process (e.g. conflict of interest related education or training for guideline panel members, teaching sessions for patients to be involved in the guideline group, etc.). (see Topics 4 & 6) |
| 9. Set a timeline for the completion of the guideline and target dates for the completion of milestones in the guideline development process. |
| 10. Determine what, if any, legal considerations are relevant for the planned guideline (e.g. reimbursement policies for orphan drugs). |
| 11. Prepare a protocol for the entire guideline that can be completed as the project progresses in order to keep the guideline development group on track, including an outline of the overall goals and objectives for the guideline, the timeline, task assignments, steps that will require documentation of decisions, and the proposed methodology for all steps (i.e. those covered in this checklist, for example the methods for forming the guideline group, selection of topics to be covered in guideline, consensus methods, consultation methods, evidence search and selection methods, etc.). |
| ***2. Priority Setting*** |
| 1. Decide on a process for priority setting of guideline topics needed and who will be responsible for directing the process (e.g. priorities set by oversight committee at headquarters of sponsoring organization, priorities referred by government ministries of health or by professional societies). |
| 2. Apply a systematic and transparent process with specific criteria for the proposal of a guideline topic during priority setting (e.g. high prevalence and burden of disease, avoidable mortality and morbidity, high cost, emerging diseases or emerging care options, variation in clinical practice, rapidly changing evidence, etc.). |
| 3. Involve appropriate stakeholders in the priority setting process and guideline topic selection (e.g. clinicians, professional societies, policymakers, payers, the public). (see Topic 6) |
| 4. Consider and decide how different perspectives about the importance and resources required for implementing the guideline recommendations will be considered (e.g. patients, payers, clinicians, public health programs). (see Topic 11) |
| 5. Search for any existing up-to-date guidelines covering the proposed topic and assess their credibility (e.g. AGREE II). Determine whether existing guideline(s) can be adapted or if a completely new guideline should be developed. (see also Topic 10) |
| 6. Discuss the need or opportunity to partner with other organizations that develop guidelines to determine whether a collaborative effort will be sought for the development of the guideline, or any part of the guideline. |
| 7. Perform a scoping exercise for the proposed guideline topic with respect to implementation issues and barriers to change (e.g. if developed the guideline is likely to improve health outcomes, implementation of healthcare recommendations is feasible, resources are available, etc.). |
| 8. Select or provide a consensus method to be used to agree on the priorities set and the guideline topic selected (e.g. voting, Delphi consensus). (see Topic 4) |
| 9. Document the priority setting process and guideline topic selected to ensure transparency. |
| ***3. Guideline Group Membership*** |
| 1. Seek multidisciplinary representation for the guideline development group, including members from the target audience, patients and carers, frontline clinicians, content experts, methodology experts, and experts in health economics, to fulfill the roles required (e.g. for the working group, guideline panel). (see also Topic 6) |
| 2. Decide on methods for recruitment and enrollment of members for the guideline development group (e.g. widespread advertising of posts, competitive appointment by interview, etc.). |
| 3. Achieve a topic-appropriate balance of expertise and adequate representation for the guideline panel (e.g. experts and primary care physicians who form the target audience, gender and geographical distribution of panel members), which may be iterative if additional members are required as the target audience and topics within the guideline are refined. (see Topic 5) |
| 4. Consider the optimum group size for the guideline development group, particularly the guideline panel (e.g. too small of a group may lack sufficient experience, content expertise and wide representation, too large of group may lack cohesiveness and effective group interaction). |
| 5. Outline roles for the guideline group members and the tasks they will be responsible for (e.g. forming a writing team, group reporter(s) to take meeting minutes and document decisions made, providing methodology consultation, conducting systematic reviews and obtaining other evidence, providing patient perspective, providing specialist clinician perspective, etc.). |
| 6. Select group leader(s), or chair(s), experienced in group facilitation, maintaining constructive dynamics, identifying and resolving conflicts, remaining neutral and objective, and having methodological expertise and content expertise. |
| 7. Document the guideline group member selection process and roles to ensure transparency. |
| ***4. Establishing Guideline Group Processes*** |
| 1. Establish how and how often communication with guideline panel members and other groups will take place, who will be responsible for making the arrangements, and consider when to deviate from this approach. |
| 2. Set expectations and awareness of the group process through an introduction, training, and support for the guideline development group members (e.g. setting ideal conditions for group discussion and decision-making). |
| 3. As part of the training for the guideline development group, ensure that group members understand what the process and proposed methods will be and that they need to be adhered to (e.g. consensus methods that may be used, anonymous or non-anonymous voting, assessment of evidence, group discussion and contributing ideas). |
| 4. Aim to set optimal conditions for group members to be provided equal opportunities to contribute and for their ideas and arguments to be given appropriate consideration (e.g. during group discussion, decision-making, and when formulating recommendations). |
| 5. Establish methods for dealing with conflict or disputes among group members and dysfunction in the group process. |
| 6. Provide opportunities for discussion and feedback about the group process throughout the guideline development project. |
| 7. Establish a method for structured and timely distribution and archiving of documents used and produced in the guideline development. |
| 8. Set a quorum for meetings (e.g. 75% of group must be present to formulate guideline recommendations), but expect that all group members attend all meetings as far as possible. |
| 9. Set or plan meeting times and locations (virtual or in-person) in advance and prepare a scope and specific agenda for each meeting. |
| 10. Keep a record of all meetings with minutes and determine whether or not to make them publically or internally available (e.g. who attended, what was the agenda, what decisions were made, what next steps will be). |
| ***5. Identifying Target Audience and Topic Selection*** |
| 1. Identify, define and/or review the primary audience (e.g. primary care physicians, health program managers) and secondary audience(s) (e.g. hospital administrators) for the guideline and determine how many audiences can be addressed with the guideline. |
| 2. Consult appropriate stakeholders about the target audience(s) identified to ensure they are applicable for the guideline topic and no relevant audience is missed. (see Topic 6) |
| 3. Establish a method and criteria to generate and prioritize a candidate list of topics to be addressed within the guideline (e.g. where evidence is most confusing or controversial, where there is currently uncertainty or inconsistency in practice, questions about screening, diagnosis, and treatment, etc.). |
| 4. Consult appropriate stakeholders to ensure all relevant topics for the guideline have been identified and will meet the needs of the target audience(s). (see Topic 6) |
| 5. Select or provide a consensus development method to be used by the group in agreeing on the final topics selected to be addressed within the guideline (e.g. Delphi method, nominal group technique). |
| 6. Document the processes of identifying the target audience(s) and selection of topics for the guideline to ensure transparency. |
| ***6. Consumer and Stakeholder Involvement*** |
| 1. Identify the appropriate stakeholders to involve and consult with in the development of the guideline to incorporate views of all those who might be affected by the guideline (e.g. professional groups, health managers, policy makers, industry representatives). |
| 2. Identify the appropriate consumers to involve and consult with in the development of the guideline (e.g. individual patients, carers who provide non-reimbursed care and support to patients, members of the public as potential patients and as funders of healthcare through taxation, community organizations that represent the interests of patients, and advocates representing the interests of patients and carers). |
| 3. Establish methods for consumer and stakeholder involvement and maintain a registry of stakeholders for the guideline (e.g. enrollment of consumer and stakeholder members to participate directly on the guideline panel, announce call for separate consumer and stakeholder meeting(s) or workshop(s), electronic distribution of documents and feedback, open period for review of documents and feedback). |
| 4. Provide information (e.g. training and introduction sessions) for consumers and stakeholders involved directly on the guideline panel to clarify roles and maximize contributions (e.g. evaluating evidence objectively, avoiding recommendations based on self-interests). |
| 5. Determine the roles, tasks and timing for consultation with consumers and stakeholders not directly participating on the guideline panel (e.g. at specific milestones during the guideline development process including opportunities to comment on priority setting, topics for the guideline, identifying target audience, identifying patient-important outcomes, identifying additional evidence, point to consequences that the panel has not considered, review the final guideline draft, etc.). |
| 6. Develop or adopt standard templates for consumer and stakeholder input and comments during consultation, with clear instructions or training modules to ensure effective input. |
| 7. Offer adequate time for consumer and stakeholder feedback and consultation. |
| 8. Set a policy and process for handling consumer and stakeholder feedback and dealing with different perspectives (e.g. ensure that diverse perspectives are taken into account in making decisions, provide transparent rationale for judgements made, provide an appeal process for stakeholders, publish consultation comments and the guideline development panel’s responses). |
| 9. Document the enrollment and selection of consumers and stakeholders for the guideline panel and the involvement and consultation with all other consumers and stakeholders to ensure explicit and transparent methods. |
| ***7. Conflict of Interest (COI) Considerations*** |
| 1. Set a policy for declaration of interests (DOI) of individual participants at admission to the project, including potential guideline panel members prior to their involvement (e.g. what interests should be disclosed, financial, intellectual, academic/clinical, competitive interests of the professional society). |
| 2. Set a policy for determination of conflicts of interest (COI) and an approach for collecting and updating COI declarations (e.g. how and what level of financial interest should be disclosed, how long a period of time should be covered by the disclosure, who will judge what constitutes a conflict). |
| 3. Provide clear instructions and training to the potential guideline group members on how to complete the COI disclosure, including a list of the members who must declare COI and the types of interests to declare including examples. |
| 4. Set a policy for management of COI (e.g. individuals with COI not categorically excluded from guideline development but excused from voting on specific recommendations related to the area of conflict, chair should have no COI, evidence summaries prepared by un-conflicted methodologists,). |
| 5. Set a policy to manage COI with respect to funding of the guideline development activities (e.g. advocate for public funding, no commercial sponsorship, commercial sponsorship from entities unrelated to topic of guideline, commercial support for non-direct activities such as translation, no single-source sponsor). |
| 6. Disclose and publish the funding source and describe the role of the sponsors and support provided for the development of the guideline. |
| 7. Explicitly disclose, publish and describe conflicts of interest of the guideline group members, particularly where the conflicts bear on specific recommendations. |
| ***8. (PICO) Question Generation*** |
| 1. Establish methods for generating the questions for the guideline, prioritizing questions, and selecting and ranking outcomes. |
| 2. Generate and document the key questions (e.g. clinical, health, policy, cost-effectiveness) to be answered in the guideline using a standard format (e.g. PICO) and determine the criteria by which the questions generated will be prioritized if it is not feasible to answer all questions (e.g. survey guideline panel members, survey stakeholders). |
| 3. Explicitly describe the population to whom the guideline is meant to apply. Take into consideration specific characteristics of the population, such as prevalence of multiple comorbidities in the population, geographical setting, and equity issues (e.g. plausible reasons for anticipating differential relative effects across disadvantaged and advantaged populations). |
| 4. Determine if regulatory approval is a requirement or not for considering interventions (e.g. for international guidelines this may be not relevant as regulatory approval may not be present for all target countries). |
| 5. Explicitly describe the intervention(s) and comparator(s) to be considered in the guideline and develop an analytic framework depicting the relationships among interventions and outcomes. Identify whether or not multiple (treatment) comparisons should be included. |
| 6. Identify the important outcomes (e.g. outcomes along the clinical pathway; morbidity, quality of life, mortality), including both desirable (e.g. benefits, less burden, savings) and undesirable effects (e.g. harm, burden, costs, and decrease in patient autonomy). Do not ignore important outcomes for which evidence may be lacking, |
| 7. Determine the setting (e.g. countries, hospitals) or include it in the considerations about the population (i.e. population cared for in tertiary care hospitals). |
| 8. Mandate a preference for patient-important outcomes over surrogate, indirect outcomes. Consider appropriateness of surrogate outcomes along the causal pathway when data for a patient-important outcome is lacking. |
| 9. Rank the relative importance of the outcomes, taking into consideration the values and preferences of the target population. |
| 10. Determine or develop a process for determining a priori the magnitude of effect for the individual outcomes that is judged as important to the target population. |
| 11. Involve all guideline group members and consult consumers and stakeholders to ensure broad representation from the target population in generating the questions and selecting and rating the important outcomes. |
| 12. Document the methods of question generation and prioritization, selection and ranking of outcomes, and stakeholder and consumer consultation to ensure they are explicit transparent. |
| 13. Ensure the guideline protocol outlines the target population, target condition, outcomes, and key questions considered to help direct the evidence review. |
| ***9. Considering Importance of Outcomes and Interventions, Values, Preferences and Utilities*** |
| 1. Decide whether the relative importance of outcomes and interventions, values, preferences or utilities of consumers and stakeholders (e.g. patients and target audience) to inform decisions and deliberations during the guideline development will be elicited indirectly or directly (e.g. review of the published literature vs. consultation with consumers). |
| 2. Establish methods for consultation with consumers and stakeholders to obtain information about the relative importance of outcomes and interventions, values, preferences or utilities (e.g. involvement of consumers on guideline panel, surveys or focus groups with broader representation of consumers). |
| 3. Determine if a structured approach for assessing the confidence in the obtained importance ratings, values, preferences and utilities (i.e. quality of the evidence in them) will be used. |
| 4. Determine if modelling will be used to integrate the relative importance of outcomes and interventions, values, preferences or utilities and how modelling will be done. |
| 5. Determine whose perspective(s) will be considered when obtaining information about the relative importance of outcomes and interventions, values, preferences or utilities and when making decisions or formulating recommendations (e.g. patients, public, society, clinicians). |
| 6. Consider and document approaches for dealing with conflicting relative importance ratings for outcomes and interventions, values, preferences or utilities (e.g. patient vs. carer, patient vs. public). |
| 7. Document the methods of obtaining information about the relative importance of outcomes and interventions, values, preferences or utilities to ensure they are explicit and transparent. |
| 8. Document if ethical considerations, such as whether recommendations should give special consideration to certain patient groups or conditions (e.g. elderly, rare disease, those affected by health inequalities). |
| 9. Decide how to consider ethical or moral values in making healthcare recommendations (e.g. by considering religious, social, or cultural convictions). |
| ***10. Deciding what Evidence to Include and Searching for Evidence*** |
| 1. Follow systematic review methods (either full systematic reviews or rapid systematic reviews depending on the topic and organization’s framework) or provide a rationale for why this is not done. |
| 2. Develop a protocol for locating, selecting, and synthesizing the evidence (e.g. conduct a search for existing systematic reviews, new systematic review and grey literature search) and determine the types of evidence to include (e.g. databases searched, types of studies, inclusion and exclusion criteria, searching for specific studies on adverse effects or deciding to abstract information on adverse effects from studies on benefit). |
| 3. Decide who will develop the search strategies and perform searching and selection of evidence (e.g. working group of guideline development group, outsource to external agency, form a relationship between guideline development group and external agency to collaborate on development of the guideline). |
| 4. Critically appraise existing systematic review(s) selected to be included using a validated tool (e.g. AMSTAR) to ensure it is of adequate quality and appropriate for use in the guideline. |
| 5. If an existing systematic review is updated or requires updating, determine how new evidence will be included and how those who conducted the review will be contacted and possibly involved in the update. |
| 6. If a new systematic review is required, conduct an assessment to determine if adequate resources (e.g. time and funding) are available to conduct a full systematic review. |
| 7. If resources are limited, consider applying a rapid assessment methodology and explicitly describe the methodology, noting important limitations, uncertainties, and the need and urgency to undertake a full systematic review. |
| 8. Establish methods for identifying additional evidence and unpublished data (e.g. suggestions from guideline panel members, consulting with stakeholders). |
| 9. Set a policy for handling expert input (i.e. expert opinion is not evidence per se and should not be used as evidence; rather, experience or observations that support expert opinions should be described, identified and, if possible, appraised in a systematic and transparent way, e.g. in the conceptual framework). |
| 10. Document and publish the search and selection of evidence, judging eligibility, range of evidence included, and search strategies used to ensure the methods are explicit and transparent. |
| ***11. Summarizing Evidence and Considering Additional Information*** |
| 1. Summarize the evidence using a concise summary (e.g. evidence table, evidence profile or summary of findings table) of the best available evidence for each important outcome, including diagnostic test accuracy, anticipated benefits, harms, resources (costs), the quality of evidence rating, and a summary of the relative and absolute results/estimate of effect for each outcome. |
| 2. Provide a summary of the additional information needed to inform recommendations (e.g. qualitative narrative summary, evidence table), including values and preferences, factors that might modify the expected effects, need (prevalence, baseline risk, or status), effects on equity, feasibility, and the availability of resources. |
| 3. Establish methods for obtaining information about resource use and cost (e.g. searching for existing economic evaluations, developing economic model, performing cost-effectiveness analysis). |
| 4. Identify the costs, resource use, and, if applicable, cost-effectiveness and describe the nature of the costs (patient, community, society) (e.g. affordability considerations, estimates of resource use and acquisition costs weighed directly against evidence of benefits and harms of an intervention). |
| 5. Document the methods in which the additional information is to be incorporated with the synthesized evidence to ensure transparency (e.g. formal consensus on patient values, consensus on equity issues, formal economic analysis, consideration of disaggregated resource use data in a qualitative manner,). |
| 6. Provide training about the use of the evidence tables and opportunities for discussion to ensure all members of the guideline panel are familiar with the tables and use them in the appropriate manner. |
| 7. In addition to the evidence summary, make available the full systematic review(s) and the original studies and other sources of evidence for the guideline panel to inform deliberations (e.g. set up a collaborative website and/or make available at meetings and via electronic communication). |
| ***12. Judging Quality, Strength or Certainty of a Body of Evidence*** |
| 1. Select a framework outlining the criteria to be considered in rating the quality of evidence (e.g. GRADE, USPSTF). Avoid modifying grading tools. |
| 2. Decide who will be responsible for appraising the quality of evidence (e.g. un-conflicted methodologists participating in the working group). |
| 3. Assess the quality of evidence for each important outcome. |
| 4. Assess the overall quality of evidence (e.g. lowest quality of evidence from outcomes rated as most important or critical, or highest quality of evidence when all outcomes point in the same direction). |
| 5. Report the quality of evidence assessed for the outcomes and the body of evidence. |
| 6. Document the judgements made in appraising the quality of evidence to ensure they are transparent and explicit. |
| ***13. Developing Recommendations and Determining their Strength*** |
| 1. Apply a framework outlining the factors to be considered to arrive at a recommendation. |
| 2. Plan and share the logistical details of the consensus meeting(s) during which recommendations will be formulated with the participants, including distribution of documents required for the meeting (e.g. evidence summaries, evidence-to-recommendation tables), setting an agenda for the meeting(s) and selecting a consensus development method to be used by the group in agreeing on judgements (e.g. Delphi method, nominal group technique). |
| 3. Review the factors of the framework that influence the recommendation, including the direction and strength (e.g. the types of evidence and information relevant to the analysis focusing on the balance between desirable and undesirable consequences informed by the quality of evidence, magnitude of the difference between the benefits and harms, the certainty about or variability in values and preferences, resource use, equity and other factors). |
| 4. If applicable, make provisions for formulating recommendations in situations where there is insufficient evidence or very low quality evidence (e.g. conditional recommendation with judgements laid out transparently, no recommendation if the guideline panel feels there is substantial risk that their decision may be wrong, recommend that the intervention be used in the context of research complemented by guidance for what are the best management options until further research becomes available). |
| 5. Make provisions for formulating research recommendations and decide where to report them (e.g. in the guideline appendix, suggesting the specific research questions, specific patient-important outcomes to measure and other relevant aspects of what research is needed to reduce the uncertainty about the benefits and/or undesirable downsides of the intervention). |
| 6. Formulate the recommendations and summarize the rationale for each recommendation (e.g. narratively or in a table), including details about the judgements made by the group and the explicit link between the recommendation and evidence supporting the recommendation. |
| 7. Select a method for rating the strength of the formulated recommendations to inform the audience of the guideline about the degree of the guideline group’s confidence about following that recommendation. |
| 8. Select a consensus development method to be used by the group in rating the strength of recommendations (e.g. Delphi method, nominal group technique, voting). |
| 9. Provide suggestions about whether the recommendations are appropriate to serve as performance measures/quality criteria (e.g. management options associated with strong recommendations based on high- or moderate-quality evidence are particularly good candidates for quality criteria, when a recommendation is weak, discussing with patients the relative merits of the alternative management strategies and appropriate documentation of this interaction may become a quality criterion). |
| 10. Document the judgements made in formulating the recommendations and determining their strength to ensure they are transparent and explicit. |
| ***14. Wording of Recommendations and of Considerations of Implementation, Feasibility and Equity*** |
| 1. Decide on standardized wording to use for recommendation statements to ensure clarity and to maintain consistency throughout the guideline, avoiding wording that may be vague and nonspecific. |
| 2. Write the recommendations in a way that is actionable with sufficient information so that it is not necessary for guideline users to refer to other material in order to understand the recommendation. |
| 3. Provide clear direction or an interpretation aid to describe the implication of the strength of recommendation for clinicians, patients, policy makers, and any other target audience groups. |
| 4. Indicate in the recommendation statements the population for which the recommendation is intended, the intervention being recommended, and the alternative approach(es) or intervention(s). |
| 5. Include remarks that describe the context, feasibility and applicability of the recommendation and highlight key considerations such as equity issues and specific conditions that might apply to the recommendation (e.g. whether the conditions outlined apply to a specific subpopulation, specific types of the intervention, for certain values and preferences, when certain resources are available, etc.). |
| 6. Report the quality of evidence and the strength of recommendation in proximity to the recommendation statement. |
| 7. Establish methods to be used by the group in agreeing on the final wording of recommendation statements (e.g. review and approval, formal consensus). |
| 8. Report the recommendations in a way that is comprehensible and visible (e.g. do not embed recommendations within long paragraphs, group recommendations together in a summary section). |
| ***15. Reporting and Peer Review*** |
| 1. Develop or adopt a standardized format for reporting the guideline, with specific structure, headings, and content. |
| 2. Decide on the format(s) to be prepared for the guideline product(s) (e.g. full guideline, full guideline with technical report/systematic reviews, brief guideline for clinicians or policymakers, consumer version for patients) that will correspond to the dissemination plan. (see Topic 16) |
| 3. Decide who will be responsible for writing the guideline product(s) (e.g. sub-committee of the guideline working group) and decide on authorship (e.g. individual authors, organization as author, working group as author). (see Topic 1) |
| 4. Conduct a review of the final draft of the guideline report(s) by all members of the guideline development group, allowing sufficient opportunity for feedback, editing and revisions. |
| 5. Seek approval from all members of the guideline development group for the final document(s). |
| 6. Initiate organizational (i.e. internal) peer review. |
| 7. Decide on the method(s) of external peer review, to review the final document(s) for accuracy, practicality, clarity, organization, and usefulness of the recommendations, as well as to ensure input from broader and important perspectives that the guideline group did not encompass (e.g. invited peer review, public consultation period with incorporation of feedback and responses from the guideline development group, submitting to peer-reviewed publication). |
| 8. Document the internal and external peer review process and, if applicable, publish consultation comments and the guideline development group’s responses. |
| ***16. Dissemination and Implementation*** |
| 1. Prepare an active dissemination plan with various approaches to enhance the adoption of the guideline (e.g. make guideline available online, develop formal relationships with those in health care systems responsible for guideline dissemination and implementation to support guideline uptake, press conference, social media strategy, dissemination at professional society meetings, publish guideline in a journal that is accessed by the target audience). |
| 2. Develop or adapt tools, support, and derivative products to provide guidance on how the recommendations can be implemented into practice (e.g. mobile applications, integration with clinical decision support systems, make guideline adaptable as an educational resource for target audience for education outreach). |
| 3. Make considerations for adaptation of the guideline and provide specific instructions for how target end users who would like to adapt the guidelines to other contexts can do so in a systematic and transparent way (e.g. modifying a recommendation based on local resources and baseline risk, implications that deviate from the judgements made by the guideline panel). |
| 4. Set rules and regulations for translation of the guideline into other languages (e.g. allow translation by third party organizations following approval by the guideline group, include staff responsible for translation in guideline working group). |
| ***17. Evaluation and Use*** |
| 1. Conduct an internal evaluation (i.e. self-assessment) of the guideline development process, including the guideline panel meeting(s) held to formulate recommendations, by asking guideline group members for feedback. |
| 2. Consider pilot testing the guideline with the target end users (e.g. with members of target audience and stakeholders who participated in the guideline development group). |
| 3. Provide criteria and tools for target end users to monitor and audit the implementation and use of the guideline recommendations (e.g. identify outcomes that should change with implementation and suggest methods for measuring the outcomes). |
| 4. Provide support and tools for prospective evaluation of the guideline to determine its effectiveness after implementation (e.g. using randomized evaluations where possible, using before-after evaluations cautiously due to uncertainties regarding the effects of implementation). |
| 5. Consider the potential involvement of the guideline development group in prospective evaluation(s) of the guideline (e.g. partnering with organizations that implement the guideline to plan evaluation studies). |
| 6. Plan to collect feedback and evaluations from users to identify how to improve the intrinsic implementability of the recommendations in subsequent versions of the guideline. |
| ***18. Updating*** |
| 1. Set a policy, procedure and timeline for routinely monitoring and reviewing whether the guideline needs to be updated (e.g. update systematic review every 3 years to determine if there is any new evidence available). |
| 2. Decide who will be responsible for routinely monitoring the literature and assessing whether new significant evidence is available (e.g. consider involvement of experts not previously involved in the guideline development group to periodically review the guideline). |
| 3. Set the conditions that will determine when a partial or a full update of the guideline is required (e.g. if only certain recommendation statements need to be updated, or whether many recommendations are out of date making the entire guideline invalid, or when recommendations are necessary for newly available treatments). |
| 4. Make arrangements for guideline group membership and participation after completion of the guideline (e.g. rotating membership every 1-2 years, selection of a new group at time of updating, continuing participation by guideline panel chair). |
| 5. Plan the funding and logistics for updating the guideline in the future (e.g. securing ongoing funding, standing oversight committee to oversee the updating process). |
| 6. Document the plan and proposed methods for updating the guideline to ensure they are followed. |
